# Smoking and Predictors of Pneumonia Among HIV-Infected Patients Receiving Care in the HAART Era

**DOI:** 10.2174/1874306400802010022

**Published:** 2008-02-26

**Authors:** David M Murdoch, Sonia Napravnik, Joseph J Eron Jr, Annelies Van Rie

**Affiliations:** 1 Division of Pulmonary & Critical Care Medicine, Duke University Medical Center, Durham, North Carolina; 2 Department of Epidemiology, School of Public Health, University of North Carolina at Chapel Hill, Chapel Hill, North Carolina; 3 Division of Infectious Diseases, School of Medicine, University of North Carolina at Chapel Hill, Chapel Hill, North Carolina, USA

## Abstract

Background: Smoking tobacco is disproportionably common among HIV-infected patients in the highly active antiretroviral therapy (HAART) era. Methods: An observational cohort study of 300 HIV-positive patients receiving care between 1996 and 2005 examined the effect of smoking on pneumonia risk. Multivariable analyses assessed the association between smoking and pneumonia risk and identified independent predictors of pneumonia during the HAART era. Results: Current smoking was common (67%). Eighty-two patients (27%) experienced 119 pneumonia episodes during 2151 patient-years of follow-up, with 7.2 episodes/100 person-years among smokers and 2.9 episodes/100 person-years among non-smokers (unadjusted incidence rate ratio (IRR): 2.50 (95% CI: 1.58, 4.09). Adjustment for age and HIV RNA level resulted in an IRR of 1.77 (95% CI: 0.98, 3.21). No prior antiretroviral therapy use (P-value <0.001), higher HIV RNA level (P-value = 0.01), lower CD4 count (P-value = 0.01), younger age (P-value = 0.01), and alcohol use (P-value = 0.04) were independent predictors of pneumonia. HAART use decreased pneumonia risk (IRR 0.28, 95% CI: 0.18, 0.44). Conclusions: While HIV-positive smokers had over a 2-fold increase in the rate of pneumonia, the trend did not reach statistical significance in multivariable models. Clinical factors such as HAART, alcohol use and immunological status are important in pneumonia risk.

## INTRODUCTION

Smoking tobacco is a major public health concern, with numerous adverse health effects, including increased risks of lung cancer, heart disease, peripheral vascular disease, laryngeal cancer, oral cancer, esophageal cancer, chronic obstructive pulmonary disease, and respiratory infections [1]. In the US, smoking is responsible for nearly 430,000 deaths each year [1, 2]. Although smoking has been declining in the general population [3], tobacco smoking among people living with HIV remains high with estimates ranging from 39-70% [4-9].

Prior to the availability of highly active antiretroviral therapy (HAART), smoking increased the risk of bronchitis, hairy leukoplakia, oral candidiasis, oral warts, bacterial pneumonia, and pneumococcal bacteremia [6, 10-13], but did not appear to affect progression to an AIDS defining clinical condition or mortality [4, 14-18]. Even though pneumonia continues to be an important clinical condition among HIV-infected patients receiving HAART [19, 20], the effect of smoking on respiratory morbidity since the availability of potent antiretroviral combination therapies remains largely unknown. Additionally, few studies have focused on identifying additional risk factors for pneumonia in HIV-infected patients exclusively during the HAART era. Therefore, we conducted the present study among HIV-infected individuals receiving HIV care at a tertiary hospital in the Southeastern United States between January 1, 1996 and September 1, 2005 to: (1) assess the association between smoking tobacco and the risk of developing pneumonia; and (2) identify independent predictors of developing pneumonia.

## MATERIALS AND METHODOLOGY

### Study Population

Patients were eligible to participate if they were enrolled in the University of North Carolina Center for AIDS Research (UNC-CFAR) HIV Clinical Cohort Study, completed a comprehensive in-person interview, the Clinical Socio-Demographic and Behavioral Survey (CSDBS), and received HIV care between January 1, 1996 and September 1, 2005. Data collection for the observational clinical cohort study include electronic transfer of institutionally available data and periodic comprehensive standardized medical record reviews [21]. The CSDBS asks patients about social, demographic, and behavioral factors generally not consistently available in medical records, including detailed smoking history.

### Pneumonia

For patients completing the CSDBS, the UNC-CFAR database was interrogated to identify all pneumonia events among study participants through abstraction of chart diagnoses.A retrospective chart review was then performed for all pneumonia events and classified by a pulmonary physician (DMM). Bacterial pneumonia was classified as “probable” or “confirmed.” Probable bacterial pneumonia required: (1) a medical diagnosis of pneumonia, including at least three clinical signs and symptoms (e.g., temperature>38.0°C, heart rate >100 beats/minute, respiratory rate >22 breaths/minute, consolidation on exam, cough, fever, shaking chills, pleuritic chest pain, and/or shortness of breath); (2) a chest radiograph with a new infiltrate; and (3) a positive clinical response to antibiotic therapy. Confirmed bacterial pneumonia required the above criteria and isolation of an organism by sputum Gram stain and culture, diagnostic bronchoalveolar lavage (BAL), positive blood culture, or a positive *Streptococcus pneumoniae *or *Legionella pneumophila* urine antigen.

Probable and confirmed diagnoses of *Pneumocystis jirovecii *pneumonia (PCP) were defined as for bacterial pneumonia above in the appropriate clinical setting, with confirmed PCP requiring evidence of *P. jirovecii* on microscopic examination of BAL or sputum specimens. A pulmonary mycobacterial infection was defined as a compatible clinical and chest radiograph presentation with isolation and culture of a typical respiratory mycobacterium from two or more sputum cultures or isolation in BAL or bronchial washings warranting appropriate anti-mycobacterial therapy.

### Smoking

Since the immunological effects of smoking on the respiratory tract are likely not permanent, we elected to use current smoking and ignore history of smoking. Current smoking was assigned based on the self- reported current smoking reported at the time of the CSDBS interview. We subtracted the duration of smoking (pack-years) from the CSDBS interview date to avoid misclassification. Since patients contributed time before and after the CSDBS interview, and because the median number of years smoking in the study population was 20 years (Interquartile range (IQR): 15, 28), we carried the latest smoking status forward.

### Covariates

For the first aim of this study we considered a number of clinical and demographic characteristics as possible effect measure modifiers or confounders of the association between smoking and risk of pneumonia. Covariates included sex, race, age, intravenous drug use (IVDU), alcohol abuse, crack cocaine abuse, vaccination history, HAART use, CD4 cell counts, and HIV RNA levels. Vaccination was defined as receiving an *influenza* vaccine within the prior one year or a pneumovax vaccine in the prior five years [22]. Intravenous drug use (IVDU), crack cocaine use, and alcohol abuse were based on self-reported behavior on the CSDBS and medical record reviews. These same factors were also included in the second aim of this study to identify independent predictors of pneumonia incidence.

### Statistical Analyses

Patient follow-up (person-time at risk) began on the latter of January 1st, 1996 or the date of entry to HIV care, and ended on the earlier of September 1st, 2005 or the last HIV clinic visit. All factors which vary over time were calculated relative to study entry, each pneumonia event, and end of follow-up. Crude incidence rates for pneumonia were calculated by dividing the number of pneumonia events by person-time at risk and expressed as number of pneumonia cases per 100 person-years.

Demographic and clinical characteristics were contrasted by current smoking status relying on basic descriptive statistics, including the chi-square test for categorical variables and the Mann-Whitney test for continuous variables. Stratified analyses included calculating incidence rate ratios (IRR) and 95% confidence intervals (CI) for the effect of smoking on the rate of pneumonia within different strata of patient characteristics. Since each patient could have experienced more than one pneumonia event during follow-up, we fit multivariable Poisson distribution log-linear regression models with generalized estimating equations (exchangeable working correlation matrix) to account for repeat measures.

The first aim of this study was to arrive at the least biased estimate of the effect of smoking on the incidence rate of pneumonia in a subset of HIV-infected patients in the UNC CFAR cohort. We first fit a full model containing all factors identified in stratified analyses as either potential effect measure modifiers or confounders for the association between smoking and pneumonia. Interaction terms with P-values ≤0.20, indicated effect measure modification. Using a hierarchical backwards elimination procedure, covariates which changed the unadjusted estimate by at least 10%, indicating appreciable confounding, were retained in the final model. The 10% change-in-estimate was calculated as ln | IRR_FULL_ / IRR_REDUCED_ |.

The second aim of this study was to estimate independent predictors of acquiring pneumonia. We first fit a full model including all covariates that were associated with pneumonia as indicated by a P-value <0.05 in bivariate analyses. Factors which did not independently predict pneumonia based on an *a priori *significance level of <0.05 were removed using a hierarchical backwards elimination procedure. Only those variables with P-values <0.05 were retained in the final predictive model. All analyses were performed using STATA version 8.2 (College Station, Texas, USA). The study was reviewed and approved by the institutional review board.

## RESULTS

### Study Population

Between January 1996 and September 1, 2005, 1849 patients were enrolled in the UNC-CFAR Clinical Cohort Study. Among these, 303 completed the CSDBS interview. We excluded 3 patients because of missing CD4 cell counts or HIV RNA levels. The 300 patients included in this study contributed 2151 person-years of follow-up during the study period, for a median of 6.9 years of follow-up [IQR: 4.6,9.2]. Two-thirds were men (67%), 76% were African American and 24% White. The median age was 44 years (IQR: 38, 48). Substance abuse was common, with a high proportion of patients reporting prior and/or current alcohol abuse (60%), crack-cocaine use (73%), or IVDU (20%). During follow-up almost all patients received antiretroviral therapy (83%) and 55% received an influenza and/or pneumococcal vaccine. With the exception of a longer duration since HIV diagnosis, the CSDBS subjects in this study are clinically and demographically representative of the overall UNC CFAR cohort [21].

### Pneumonia Diagnosis

Eighty-two patients (27%) were diagnosed with 119 episodes of pneumonia for an overall incidence rate of 5.5 per 100 person-years (95% CI: 4.6, 6.6). The majority of pneumonias were bacterial (61%), with Streptococcus pneumoniae being the most commonly isolated organism (Table **1**). The AIDS-defining diagnosis of PCP accounted for over one-third (34%) of pneumonia diagnoses. Of the PCP cases, 44% were on HAART at diagnosis. The diagnosis of mycobacterial infections in this cohort was uncommon, representing only 4.2% of pneumonia diagnoses.

Fifty of the 119 pneumonia events (42%) were confirmed, including 56% of PCP cases, 35% of bacterial pneumonia cases, and all mycobacterial cases. At the time of the pneumonia event the median CD4 cell count was 193 cells/mm^3^ (IQR: 35, 424) among patients with bacterial pneumonias and 35 cells/mm^3^ (IQR: 13, 118) among patients with PCP (P-value < 0.001).

### Smoking Prevalence and Characteristics of HIV-Infected Smokers

Most patients (N=241, 80%) reported ever having smoked tobacco for at least six months, and reported a median of 20 years of smoking (IQR: 15, 28), 10 cigarettes per day (IQR: 5, 20), and 10 pack-years of smoking exposure (IQR: 5, 20). At the time of the interview 63% (N=188) reported currently smoking tobacco. On bivariate analysis, smokers were more likely to be male, to use other substances of abuse such as alcohol, intravenous drugs, and cocaine (Table **2**). Additionally, median CD4 cell counts were lower in smokers compared to non-smokers (351 cells/mm^3^ versus 490, respectively; P-value = 0.01) and HIV RNA levels were higher (2.8 log_10_ copies/mL versus 1.9, respectively; P-value = 0.01).

### Effect of Smoking on Pneumonia Incidence Rates

The majority of pneumonia episodes occurred among smokers (95 of 119; 80%). The incidence rate of pneumonia among smokers was 7.2 per 100 person-years (95% CI: 5.8, 8.8) in comparison to 2.9 (95% CI: 1.8, 4.3) among non-smokers, for an unadjusted incidence rate ratio (IRR) of 2.5 (95% CI: 1.58, 4.09). Effect measure modifiers were not identified. Age and most proximal HIV RNA level were the only confounders of the association between smoking and pneumonia incidence, and were retained in the final model. The adjusted incidence rate ratio for smoking was 1.77 (95% CI: 0.98, 3.21; P-value = 0.06) (Table **3**).

### Independent Predictors of Pneumonia

For the second aim, a number of patient characteristics were predictive of developing pneumonia in bivariate analyses, including smoking, African American race, younger age, alcohol use, crack cocaine use, antiretroviral use, a lower CD4 cell count and higher HIV RNA level (Table **4**). In multivariable analyses, younger age, alcohol use, lack of antiretroviral use, a lower CD4 cell count, and a higher HIV RNA were identified as independent predictors of pneumonia among all patients (Table **5**). Antiretroviral therapy use was highly protective of developing pneumonia (IRR 0.28). CD4 cell count was inversely associated with pneumonia incidence (for each 100 CD4 cell count increase the incidence rate decreased by 16% (IRR = 0.84). HIV RNA level was directly associated with an increase in pneumonia incidence rate (1.52 fold increase for each 1 log_10_ HIV RNA level increase). Consistent with the first study aim, smoking increased the risk of pneumonia but the trend did not reach statistical significance in the multivariable model (IRR 1.77, 95% CI: 0.99, 3.15).

## DISCUSSION

We observed a high burden of pneumonia among people living with HIV and receiving care in the Southeastern US between 1996 and 2005. More than one in four (27%) patients had at least one pneumonia episode during an average of seven years of follow-up.

The majority of pneumonias were bacterial (61%), but a substantial number (35%) were PCP. Consistent with other studies, we observed an extremely high burden of tobacco use in this cohort. This observation suggests that, despite advances in HIV care [6, 23], respiratory infections continue to be a problem among people living with HIV in the US and tobacco use is disproportionably high in this population.

The majority (80%) of pneumonia episodes occurred among smokers. The estimated incidence rate of pneumonia among smokers was 7.2 per 100 person-years versus 2.9 in non-smokers. Smoking is reported to be associated with an increased risk of pneumonia in HIV-infected patients prior to the use of potent combination antiretroviral therapies [13, 14] and in studies that span the pre-HAART and HAART eras [24]. The lack of association between smoking and pneumonia observed in this study may be a result of patients exclusively from the HAART era, the high proportion (83%) of HAART use, the relatively small sample size, and the small proportion of never smokers in this cohort. Additionally, the inclusion of PCP, which may be affected more by CD4 cell counts and HAART use rather than by smoking, may have affected the observed association. However, even though smoking was not an independent predictor of pneumonia in this study population, the two fold increase in incidence rates combined with the extremely high prevalence of smoking suggests that smoking cessation should be an important intervention to reduce the burden of pneumonia among people living with HIV, particularly since smoking negatively impacts on morbidity and mortality in this population [23].

The increased risk of pneumonia in smokers may be explained by a number of studies of smoking in both immunocompetent and HIV-infected individuals. In immunocompetent individuals, smoking impairs ciliary function [25], which may result in pathogen colonization and airway inflammation [26, 27]. In addition, nicotine has been shown to suppress leukocyte migration, and cigarette smoke is directly cytotoxic to alveolar type II cells [28, 29]. Compared to HIV-infected non-smokers, HIV-infected smokers demonstrate decreased levels of secreted proinflammatory cytokines interleukin-1 (IL-1) and IL-6 by lung macrophages [30] and reduced concentrations of IL-1β and TNF-α in BAL fluid [31]. Furthermore, alveolar macrophages recovered from HIV-positive smokers demonstrate decreased phagocytic function to both *Escherichia coli* bacteria and IgG-opsonized sheep red blood cells compared to HIV-infected non-smokers [32]. These data may partially explain why smoking as an independent risk factor for invasive pneumococcal disease in HIV-infected and uninfected cohorts [12, 33].

To better understand how the burden of pneumonia in the HAART era could be further reduced, the second study aim identified independent predictors other than smoking for developing pneumonia among people living with HIV. Antiretroviral use was highly protective and, consistent with previous observations [13, 14, 19, 20, 24], a low CD4 cell count was significantly associated with pneumonia risk. These data indicate the importance of early diagnosis of HIV infection and access to care prior to the development of severe immunosuppression. In contrast to previous observations [13, 19, 20, 34], intravenous drug use was not predictive of pneumonia in bivariate or multivariate analyses, possibly as a result of the relatively low use in this cohort. Younger age was predictive of pneumonia risk, which may be partially explained by early AIDS-related diagnoses at entry to HIV care in this population [35]. Interestingly, alcohol use was identified as a predictor of pneumonia in the predictive modeling analyses. This observation is important because alcohol use among HIV-infected patients is high, is often associated with other substance abuse [36, 37], and is associated with pneumonia in HIV-uninfected cohorts [38].

This analysis was limited by several factors. First, the diagnosis of pneumonia was made as part of routine care, and without bacteriological confirmation in 53% of cases. Increased rates of bronchitis and non-specific respiratory complaints in smokers may have led to misclassification of these subjects as pneumonia events. However, the use of chest radiographs minimized this bias. Second, this analysis relied on self-reported behaviors during the CSDBS interview. However, based on experiences in other studies, the self-reported current smoking status and amount of smoking measured in pack-years was unlikely to be biased [39, 40]. To minimize the impact of self-reported behavior, we attempted to corroborate self-reported behaviors with clinical diagnoses. Third, we were not able to adequately control for the use of opportunistic infection prophylaxis and antimicrobial use during the observation period. Attempts to control for these factors resulted in collinearity with antiretroviral use and unstable model estimates. Lastly, subjects who completed the CSDBS questionnaire represented a subset of the overall CFAR cohort. With the exception of a longer duration since HIV diagnosis, the CSDBS subjects in this study are clinically and demographically representative of the overall UNC CFAR cohort [21].

## CONCLUSIONS

With the introduction of highly active antiretroviral therapy (HAART), significant declines in the burden of pneumonia have occurred in people living with HIV. However, despite these declines, smoking and other substance abuse remains high, with increased incidences of pneumonia among smokers and those who report alcohol use. This observation, coupled with the increased risk in those presenting with advanced immunosuppression, suggest that early access to HIV care programs incorporating substance abuse treatment programs may further reduce the burden of pneumonia among people living with HIV.

## Figures and Tables

**Table 1:** Clinical Characteristics of 119 Pneumonia Episodes Diagnosed in 82 HIV-Infected Patients, from January 1, 1996 to September 1, 2005

Total	N (%) 119 (100%)
Bacterial pneumonia Probable Confirmed *Streptococcus pneumoniae* *Legionella pneumophila* *Haemophilus influenzae* MSSA *Nocardia sp* *Pseudomonas sp* MRSA *M. tuberculosis* *M. avium-intracellulare*(MAC) *M. kansasii* *Pneumocystis jirovecii* pneumonia (PCP) Probable Confirmed DF: Done – positive* Done – negative Not done BAL cytology: Done – positive* Done - negative Not done	78 (66%) 51 (65%) 27 (35%) 10 3 3 2 2 1 1 1 3 1 41 (34%) 18 (44%) 23 (56%) 14 16 11 10 6 25

MSSA, Methicillin-sensitive *Staph aureus;* MRSA, Methicillin-resistant *Staph aureus;* DF, Direct immunofluorescence stain; BAL, Broncho-alveolar lavage;

* Includes one patient with a positive DF and BAL cytology.

**Table 2.** Demographic and Clinical Characteristics of 300 HIV-Infected Patients Stratified by Smoking Status

Characteristic*	Smokers (N = 188)	Non-Smokers (N = 112)	p-Value±
Age (yr), med (IQR) Men, n (%) Race, n (%) African American Time since HIV diagnosis (yrs), med (IQR) MSM, n (%) Alcohol use, n (%) Previous use Current use Intravenous drug use, n (%) Previous use Current use Crack cocaine use, n (%) Previous use Current use Antiretroviral use, n (%) Time on antiretroviral therapy (yrs), med (IQR) Number of prior antiretrovirals, med (IQR) Vaccination status, n (%) CD4 cell count (cells/mm^3^), med (IQR) HIV RNA level (log_10_ copies/ml), med (IQR)	44 [40, 49] 135 (72) 145 (77) 9 [7, 14] 54 (29) 89 (47) 50 (27) 40 (21) 7 (4) 105 (56) 68 (36) 156 (83) 9 [6, 11] 4 [3, 6] 105 (56) 351 [151, 630] 2.8 [1.7, 4.4]	45 [38, 51] 65 (58) 83 (74) 10 [7, 13] 34 (30) 31 (28) 11 (10) 13 (12) 0 (0) 34 (30) 12 (11) 92 (82) 9 [7, 12] 4 [3, 7] 62 (55) 490 [272, 656] 1.9[1.7, 4.1]	0.361 0.015 0.560 0.549 0.764 <0.001 <0.001 0.034 0.048 <0.001 <0.001 0.853 0.316 0.139 0.934 0.012 0.015

* All factors which vary over time were measured at the last pneumonia event among those who had pneumonia and at the last clinic visit among those who did not experience pneumonia; med, median; IQR, interquartile range; MSM, men who have sex with men; ±

± p-value for Chi-square test for independence, t-test for equality of means using pooled variance procedures, or Wilcoxon-Mann-Whitney test, as appropriate, values ≤0.05 considered significant.

**Table 3.** Multivariate Poisson Model Estimates of the Effect of Smoking on Pneumonia Incidence Rates in 300 HIV-Infected Patients Receiving Care Between January 1996 and September 1, 2005

Covariate*	IRR	95 % CI	p-Value
Current smoker	1.77	0.98, 3.21	0.060
Age, per 10 year increase	0.62	0.41, 0.93	0.019
HIV RNA level, per 1 log_10_ increase	1.96	1.64, 2.38	<0.001

* All factors which vary over time were measured at a pneumonia event among those who had pneumonia and at the last clinic visit among those who did not experience pneumonia; IRR, incidence rate ratio; CI, confidence interval.

**Table 4.** Pneumonia Incidence Rates Among 300 HIV-Infected Patients Stratified by Demographic and Clinical Characteristics

Characteristic*	Pneumonia Events	Person-Time	Incidence Rate (95% CI)	p-Value
Smoking status Nonsmoker Current smoker	24 95	833 1318	2.9 (1.8, 4.3) 7.2 (5.8, 8.8)	<0.001
Race White Racial/Ethnic Minority	14 105	546 1605	2.6 (1.4, 4.3) 6.5 (5.3, 7.9)	<0.001
Gender Female Male	33 86	665 1486	5.0 (3.4, 7.0) 5.8 (4.6, 7.1)	0.461
Age 18-35 36-44 ≥45	27 57 35	283 744 1125	9.5 (6.3, 13.9) 7.7 (5.8, 9.9) 3.1 (2.2, 4.3)	<0.001
Intravenous drug use No Yes	90 29	1726 425	5.2 (4.2, 6.4) 6.8 (4.6, 9.8)	0.214
Alcohol use No Yes	47 72	1132 1018	4.1 (3.0, 5.5) 7.1 (5.5, 8.9)	0.004
Crack cocaine use No Yes	46 73	1084 1067	4.2 (3.1, 5.7) 6.8 (5.4, 8.6)	0.010
Vaccination status No Yes	66 53	927 1224	7.1 (5.5, 9.1) 4.3 (3.2, 5.7)	0.070
Antiretroviral use No Yes	45 74	291 1860	15.5 (11.3, 20.7) 4.0 (3.1, 5.0)	<0.001
CD4 cell count (cells/mm^3^) ≥350 200-349 50-199 <50	29 13 32 45	1189 398 342 222	2.4 (1.6, 3.5) 3.3 (1.7, 5.6) 9.4 (6.4, 13.2) 20.3 (14.8, 27.1)	<0.001
HIV RNA level (copies/ml) <400 400-9,999 10,000-99,999 ≥100,000	19 18 35 47	1130.7 379.9 417.5 222.8	1.7 (1.0, 2.6) 4.7 (2.8, 7.5) 8.4 (5.8, 11.6) 21.1 (15.5, 28.0)	<0.001

* All factors which vary over time were measured at a pneumonia event among those who had pneumonia and at the last clinic visit among those who did not experience pneumonia.

**Table 5.** Independent Predictors of Pneumonia Among 300 People Living with HIV in the Southeastern US, 1996-2005

Characteristic*	IRR	95% CI	p-Value
Alcohol use	1.67	1.02, 2.72	0.04
Antiretroviral use	0.28	0.18, 0.44	<0.001
Age, per 10 year increase	0.57	0.37, 0.89	0.01
CD4 cell count, per	0.84	0.74, 0.96	0.01
100 cell/mm^3^ increase			
HIV RNA level, per 1 log_10_ increase	1.51	1.19, 1.93	<0.001

* All factors which vary over time were measured at a pneumonia event among those who had pneumonia and at the last clinic visit among those who did not experience pneumonia; IRR, incidence rate ratio; CI, confidence interval.
